# Physical-Vapor-Deposited Metal Oxide Thin Films for pH Sensing Applications: Last Decade of Research Progress

**DOI:** 10.3390/s23198194

**Published:** 2023-09-30

**Authors:** Mohammad Nur-E-Alam, Devendra Kumar Maurya, Boon Kar Yap, Armin Rajabi, Camellia Doroody, Hassan Bin Mohamed, Mayeen Uddin Khandaker, Mohammad Aminul Islam, Sieh Kiong Tiong

**Affiliations:** 1 Institute of Sustainable Energy, Universiti Tenaga Nasional, Jalan IKRAM-UNITEN, Kajang 43000, Selangor, Malaysia; kbyap@uniten.edu.my (B.K.Y.); armin.rajabi@uniten.edu.my (A.R.); camellia@uniten.edu.my (C.D.); mhassan@uniten.edu.my (H.B.M.); siehkiong@uniten.edu.my (S.K.T.); 2 School of Science, Edith Cowan University, 270 Joondalup Drive, Joondalup, WA 6027, Australia; 3 School of Engineering and Technology, Central Queensland University Australia, Melbourne, VIC 3000, Australia; 4 National Centre for Flexible Electronics, Indian Institute of Technology Kanpur, Kanpur 208016, India; dmaurya@iitk.ac.in; 5 College of Engineering, Universiti Tenaga Nasional, Jalan IKRAM-UNITEN, Kajang 43000, Selangor, Malaysia; 6 Centre for Applied Physics and Radiation Technologies, School of Engineering and Technology, Sunway University, Bandar Sunway 47500, Selangor, Malaysia; mayeenk@sunway.edu.my; 7 Department of Electrical Engineering, Faculty of Engineering, University of Malaya, Kuala Lumpur 50603, Selangor, Malaysia; aminul.islam@um.edu.my

**Keywords:** thin film, metal oxide, pH sensor, sensitivity

## Abstract

In the last several decades, metal oxide thin films have attracted significant attention for the development of various existing and emerging technological applications, including pH sensors. The mandate for consistent and precise pH sensing techniques has been increasing across various fields, including environmental monitoring, biotechnology, food and agricultural industries, and medical diagnostics. Metal oxide thin films grown using physical vapor deposition (PVD) with precise control over film thickness, composition, and morphology are beneficial for pH sensing applications such as enhancing pH sensitivity and stability, quicker response, repeatability, and compatibility with miniaturization. Various PVD techniques, including sputtering, evaporation, and ion beam deposition, used to fabricate thin films for tailoring materials’ properties for the advanced design and development of high-performing pH sensors, have been explored worldwide by many research groups. In addition, various thin film materials have also been investigated, including metal oxides, nitrides, and nanostructured films, to make very robust pH sensing electrodes with higher pH sensing performance. The development of novel materials and structures has enabled higher sensitivity, improved selectivity, and enhanced durability in harsh pH environments. The last decade has witnessed significant advancements in PVD thin films for pH sensing applications. The combination of precise film deposition techniques, novel materials, and surface functionalization strategies has led to improved pH sensing performance, making PVD thin films a promising choice for future pH sensing technologies.

## 1. Introduction

**Figure 1:**
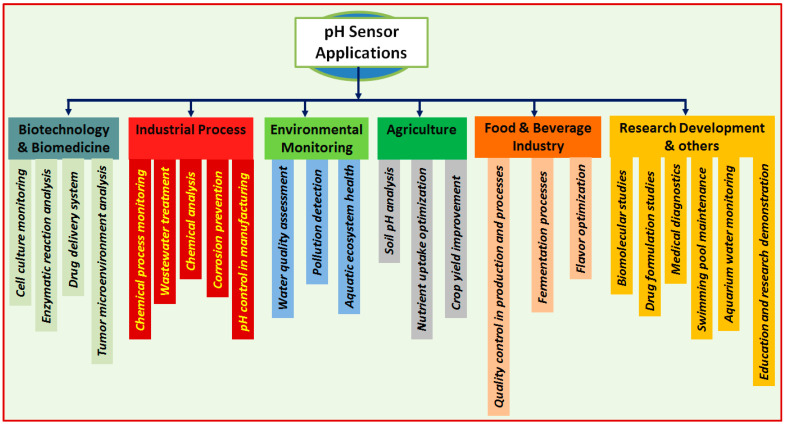
Application areas of pH sensors.

In the current nanotechnological age, pH detection has become fundamental because of its potential functionalities in different fields, providing basic data about the perceptiveness or alkalinity of an answer. Since pH is a critical component in numerous synthetic, organic, and ecological cycles, it is imperative to measure pH precisely and routinely for various applications [1,2,3,4,5]. In the areas of biotechnology and biomedicine, pH detection is fundamental for observing cell exercises, enzymatic responses, and medication conveyance systems. It allows us to upgrade and improve cell development conditions, assess the successes of remedial mediations, and explore the pH-subordinate behavior of bio-atoms. pH sensors are normally used to screen and control the pH of culture media, guaranteeing ideal circumstances for cell development and efficiency in different applications, including biopharmaceutical creation, tissue designing, and maturation processes. For instance, in malignant growth treatment, cancer microenvironments are often portrayed by acidic pH because of expanded glycolysis and lactate creation. pH sensors can be utilized to quantify and screen the pH levels in growing tissues, helping with the assessment of treatment adequacy and reaction. By following pH changes, analysts and clinicians can acquire important experiences in the adequacy of pH-delicate anticancer treatments, such as pH-actuated drug conveyance frameworks or pH-responsive imaging specialists [6,7,8,9,10]. In environmental observation, pH sensing is used to assess the strength of amphibian biological systems, evaluate water quality, and identify contamination sources. It supports the comprehension of normal cycles like corrosive downpour development, sea fermentation, and the effect of modern releases. pH detection is likewise fundamental in the food and drink industry, where pH levels determine item well-being, quality, and flavor. Observing and controlling pH during maturation processes, food safeguarding, and blending is fundamental for guaranteeing item consistency and the period of usability. Moreover, pH detection plays a crucial role in various applications, including agriculture, by enhancing soil pH levels to promote efficient nutrient uptake by plants and prevent soil degradation. It is also important in industrial contexts for process control, including chemical reactions, wastewater treatment, and erosion prevention. The efficient and cost-effective control of these operations is made possible by accurate pH assessment, assuring optimal performance and reducing environmental impact. Researchers, businesses, and conservationists can use pH detection as a useful scientific tool in a variety of disciplines to obtain knowledge, make choices, and address problems with productivity, sustainability, and health [2,8,9,10,11,12,13,14,15,16,17]. As technology continues to progress, pH sensors are becoming more advanced, accurate, and user-friendly, allowing for more precise and real-time monitoring of pH levels in various applications. One of the main drivers for the future growth of pH sensors is the increasing demand for more precise and reliable measurement techniques. Another factor is the development of new materials and technologies that enable pH sensors to be used in harsher and more demanding environments. The pH sensor market is expanding rapidly due to several key factors. For example, the food and beverage industry is increasingly focusing on ensuring food safety and quality throughout the supply chain. pH sensors play a vital role in monitoring and controlling pH levels in food and beverage processing, preserving product quality and shelf life. In emerging economies, infrastructure development, rapid industrialization, and increasing investments in sectors such as water treatment, healthcare, and manufacturing are driving the demand for pH sensors in these regions. In a healthcare context, miniaturized development of pH sensing devices and their validation with biological samples could see these devices applied in the medical field, especially in situations where current glass electrodes cannot be used due to fragility and size constraints. For example, gastrointestinal reflux disease requires pH monitoring and diagnosis is currently conducted using endoscopy. However, developing compact pH sensing devices in capsules can help retrieve data through wireless signaling from the sensor without any medical intervention to smart devices such as smartphones, which can transmit the data to a server in cloud networks for health service providers to access for their service. Figure 1 shows a schematic presentation of pH sensor application areas.

PVD thin films offer astounding sensitivity to pH changes because of their dainty and uniform construction, enabling productive interaction with the analyte for real-time, faster, and precise pH estimations that make them appropriate and attractive for dynamic pH sensing devices. Also, PVD thin films exhibit flexibility regarding substrate similarity, as they can be grown on different substrates, including adaptable and scaled-down sizes, empowering consistent coordination into small-scale devices like wearable sensors, lab-on-a-chip systems, and implantable medical devices. Furthermore, PVD methods allow precise control over material composition synthesis and thickness, leading to the testimony of a large number of materials customized for pH sensing devices and applications. This adaptability empowers scientists, specialists, and manufacturers to tailor the thin film properties, including bandgap, conductivity, structural mobility, and stability, to satisfy explicit pH-detecting prerequisites. Moreover, PVD thin films have shown strength in terms of natural factors like temperature, mugginess, and compound openness, guaranteeing dependable and durable pH sensing performance. In general, PVD thin films offer extraordinary responsiveness, fast reaction times, similarity with different substrates, adaptability in material affidavit, and long-term dependability, making them exceptionally promising for pH sensing applications [18,19,20,21,22,23,24]. However, whilst PVD thin films offer several advantages for pH sensing applications, they also have certain drawbacks that need to be considered during the development of a pH sensing electrode or a device. The disadvantages of PVD thin films for pH sensing are mainly very specific, such as deposition of a thicker film, film uniformity across large areas, material choice limitations due to deposition rate incompatibility, deposition process parameter optimization difficulties, and easy access to specialized deposition equipment and facilities. Therefore, understanding these disadvantages is crucial for researchers and engineers working on pH sensing applications using PVD thin films [25,26]. 

The goal of this review article is to deliver an inclusive synopsis of the latest developments and advancements in PVD thin films for pH sensing applications over the past decade. This work includes the exploration of PVD techniques utilized for fabricating thin films tailored for pH sensing, investigation of various thin film materials, and surface modification techniques employed for high-performance pH sensing applicable in diverse fields. Additionally, this review work also allows an understanding of the advantages, drawbacks, and limitations of PVD thin films in pH sensing, allowing identification of the challenges and future research directions in the development of PVD thin films for pH sensing applications. 

## 2. Materials and Methods

### 2.1. PVD Techniques, Advantages, and Limitations 

**Figure 2:**
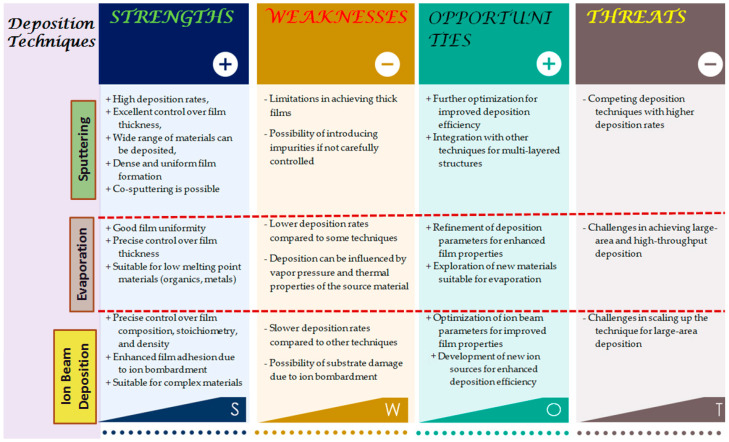
SWOT analysis of sputtering, evaporation, and ion beam deposition techniques that are commonly used for thin film preparation. Note: This SWOT chart is a synopsis of thin film deposition techniques. However, specific advantages and limitations may vary depending on the deposition system, process parameters, and materials used.

There are several physical vapor deposition (PVD) techniques that are very frequently used in thin film material synthesis and fabrication processes. Radiofrequency (RF)/DC sputtering, thermal and e-beam evaporation, ion beam deposition, pulsed laser deposition, molecular beam epitaxy, active reactive evaporation, etc., are most commonly used to develop thin film materials for many applications, including pH sensing/sensor electrode development. Each of the deposition techniques has a unique working principle. For example, sputtering requires bombarding the target material using high-energy ions, causing atoms to be ejected from the target in the form of a plasma (argon or argon plus oxygen) state and subsequently deposited onto the substrate to form a thin material layer. The main advantages of the sputtering technique include high deposition rates, excellent film thickness control, and the ability to deposit various materials such as metals, metal oxides, nitrides, and nanocomposites from the solid sputtering targets. Additionally, the sputtering technique allows the co-sputtering (deposition of materials from more than one material target at a time) process, glancing angle deposition, and the sequential deposition of different materials without changing the inside chamber environment that is suitable for various nanocomposite growth and the successful fabrication of application-specific thin film materials [19,22,23,24,27,28]. Various geometries of thin film material (single and multilayer structures deposited from metal-oxide-based iron garnet sputtering targets) fabrication processes using the sputter deposition technique have been successfully performed and showcased in the report [29]. On the other hand, the evaporation (E-beam or Thermal) method requires heating a source material with a high-energy electron beam or a high-voltage thermal unit to melt the material, vaporize it, and then allow the vapor to condense onto a substrate for thin film growth. This technique allows the size flexibility of substrates to prepare thin films with high film uniformity and precise control over film thickness. However, this technique is most fruitful for the synthesis and development of organic and metal–organic composite materials with low melting points [30,31,32,33]. Ion beam deposition utilizes an energetic ion beam to sputter atoms from a target material, which then deposit onto a substrate to grow the thin film [34]. This technique allows for precise control over film composition, stoichiometry, and film density, which is more advanced than any other thin film synthesis process. This technique is more advantageous, especially for complex material synthesis, and has been employed to deposit thin films of metal oxides as improved adhesion can be achieved due to the ion bombardment during deposition. Therefore, PVD techniques provide diverse options for designing and fabricating a range of thin films suitable for the design and development of various coatings and optoelectronic devices, such as metal–dielectric multilayer optical coatings for specific applications and metal-oxide-based electrodes for high-performance pH sensors [27]. Other fabrication methods are also reported for the growth and synthesis of thin films applicable to the development of various devices, including pH sensing components such as the spin-coating process, sol-gel, atomic layer deposition (ALD), and pulsed laser deposition techniques, which are not the main focus of this study. However, all of these commonly used techniques have some pros and cons in terms of process techniques and availability of the system. Hence, the choice of deposition technology depends on several factors such as the desired material types, film properties, and the application-specific material property requirements. A thorough description of the benefits and restrictions of each deposition method (sputtering, evaporation, and ion beam deposition) for thin film fabrication in pH sensing applications is given in the SWOT (Strengths, Weaknesses, Opportunities, and Threats) chart as can be seen in the tabulated diagram (Figure 2). 

Sputtering can deposit a variety of materials at high deposition rates with great control over film thickness, although it may have difficulty producing thick films and can introduce contaminants. The vapor pressure and temperature characteristics of the source material might affect the deposition rates, which may be lower than with other processes but still offer high layer uniformity and precise thickness control. Although ion beam deposition often has slower deposition rates and might harm substrates, it provides fine control over film composition and density, leading to improved layer adherence. The benefits of each deposition method may be increased by looking at the potential for additional optimization and integration with other methods. In general, each of these techniques has a unique set of benefits and drawbacks, and the use of methodology is completely application-dependent. However, all of these deposition techniques are suitable for depositing thin films with precise control over the film-growth process parameters that allow fine tuning of film thickness, composition, and microstructure according to application specifications, including surface modification of thin films [34,35,36,37,38,39,40,41,42,43,44,45]. Therefore, vacuum-based coating equipment is used in laboratories for cutting-edge experiments and investigations as the coating industries are demanding techniques with new working principles, and highly efficient material synthesis with more precise film quality control for the advancement of modern science and development. Therefore, researchers can choose the most appropriate deposition process for their pH sensing applications by carefully weighing these aspects.

### 2.2. Thin Film pH Sensing Material Types and Development 

**Figure 3:**
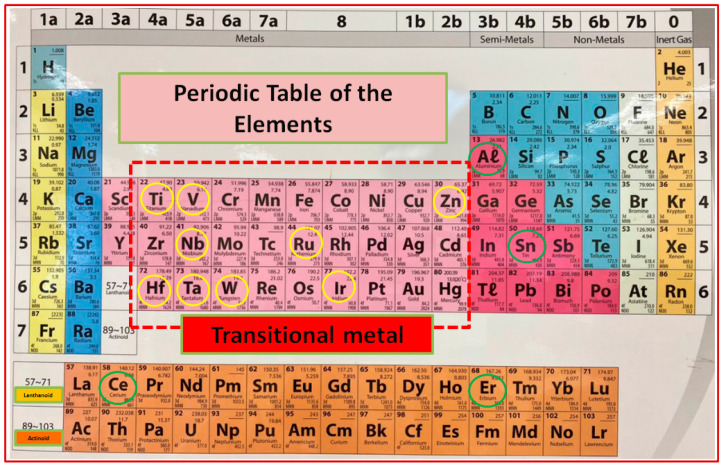
The group of materials that are frequently used to design and synthesize pH sensors/sensing devices using PVD techniques is presented in the periodic table schematically. Note that most of the metal oxides used in the last decade for pH sensor development belong to the transition metal group, as indicated by the yellow and green circles in this periodic table presentation.

The application-specific thin film pH sensors/sensor components are dependent on material selection and their fabrication technologies, including optimized process parameters. Several reports are available in the literature about various materials and compositions, including metal, metal oxides, nitrides, and nanostructured films that are commonly employed for pH sensing due to their extraordinary properties and suitability. Metal oxides, including Ruthenium oxide (RuO_2_), zinc oxide (ZnO), Titanium dioxide (TiO_2_), and Tantalum oxide (Ta_2_O_5_), have been reported by many research groups; however, Tin oxide (SnO_2_), Iridium oxide (IrO_2_), Tungsten oxide (WO_3_), Aluminum oxide (Al_2_O_3_), Erbium oxide (Er_2_O_3_), Cerium oxide (CeO_2_), and Vanadium oxide (V_2_O_5_) are also reported as pH materials in the past decade of research for the improvement of pH sensors [19,22,28,35,46]. These materials are available and easy to access, and it is possible to fine-tune their material properties to obtain high surface area and chemical and structural stability, leading excellent pH sensitivity. The physical strength of the deposited film on the substrate is mostly crucial for the development of thin-film-related optic, optoelectronic, magneto-optic, and electrochemical devices. Figure 3 represents the group of materials that are frequently used to develop pH sensors/sensing devices. 

**Figure 4:**
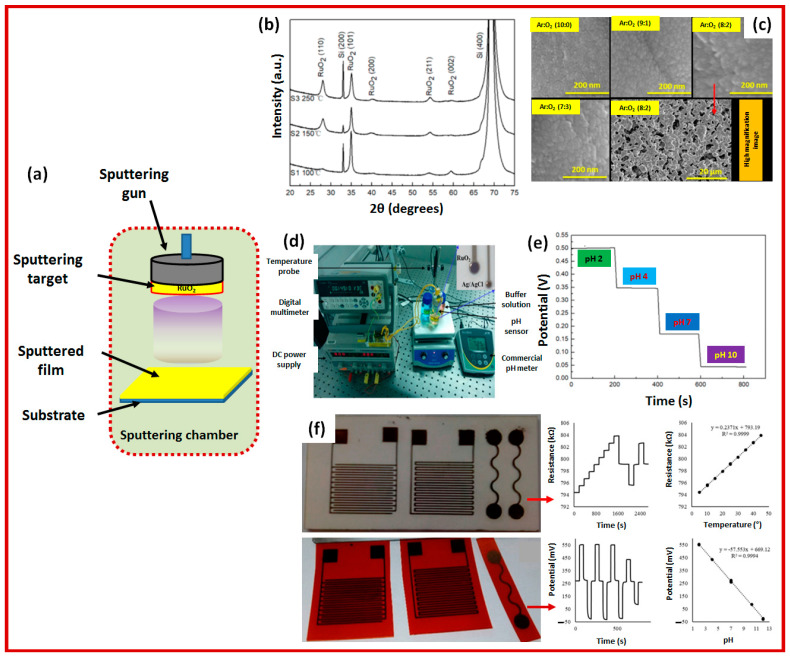
A presentation of fabrication to the characterization of RuO_2_-based pH sensing electrodes: a simple schematic diagram of a sputtering process where the material deposition process optimization was required to obtain the best material properties (**a**); the structural characterization (X-ray diffraction and scanning electron microscope image) is an essential part of developing and confirming the high-quality material layer grown (**b**,**c**); the developed material testing setup to obtain results of pH measurement (**d**); the pH stability testing result for a RuO_2_ layer (**e**); and development and testing results of RuO_2_ pH sensing electrodes on flexible substrates (**f**) [22,23,27].

The latest research history on metal oxide pH sensors revealed that the type of metal oxide used, sensing electrode thickness, deposition process parameters (depending on fabrication techniques) and the frequency range of the electrochemical impedance spectroscopy significantly affect the performance of metal oxide thin film pH sensors. A research group in Australia led by Prof Kamal Alameh reported on the study of the developments in R.F. magnetron-sputtered thin films for pH sensing applications, including the advantages of metal oxide thin film pH sensors [19,22,27,37,38]. This group has dedicated almost a decade to the development of various types of metal oxide pH sensors, including RuO_2_ pH sensing electrodes. They have studied material synthesis to experimentally test different types of pH sensors and reported that the thickness of a ruthenium oxide (prepared by RF magnetron sputtering) sensing electrode affected the measurement accuracy of the pH sensor. In addition, they have also reported on the fabrication of RuO_2_-based pH sensing electrodes on flexible substrates like alumina and polyimide for the development of less expansive pH sensing devices [27]. Another new dimension of RuO_2_-based pH sensors has been explored by developing a pH sensor with a RuO_2_ pH-sensitive working electrode and a SiO_2_-PVB junction-modified RuO_2_ reference electrode that exhibited outstanding performance, indicating a method of cost-effective, high-performance, and robust pH sensors in various applications [30]. On the other hand, another research group [36] has also studied the electrochemical reactions of conductimetric interdigitated thick film pH sensors based on RuO_2_ with solutions of different pH values, and the conductance and capacitance of the film were found to be noticeably dependent on pH in the array of low frequency. A very recent report revealed that RuO_2_ thin films sputtered from a metallic target using RF sputtering are less smooth and have high surface roughness and weak adhesion to the substrates compared to RuO_2_ films prepared by using a metal oxide target with a DC sputtering technique [23]. The conducted study investigated the effects of different magnetron sputtering conditions on RuO_2_ thin films, similar to other scientific reports [19,22,28]. However, the obtained results in this work confirmed that sputtering from metallic cathodes can result in rougher films due to oxidation reactions, while sputtering from oxide cathodes produces harder films. Higher substrate temperatures led to rougher and denser films, and lower sputtering pressures resulted in films with higher hardness and compressive residual stress, thus confirming that the DC-sputtered thin films exhibited higher pH sensitivity than RF-sputtered films. In addition, depositing durable metallic RuO_2_ thin films for steadfast sensors necessitates a chamber pressure of ≥4 mTorr with argon–oxygen content, while the choice of deposition process (pure argon plasma or argon–oxygen plasma mix) significantly impacts the resulting oxide material properties due to oxygen loss during sputtering, which can be mitigated by high-temperature oven annealing [27]. Figure 4 presents the latest updates on the development of RuO_2_ thin films for pH sensing and achieving higher performance.

ZnO is also a high-potential material for the development of pH sensors and has a long history of using various physical vapor deposition techniques. ZnO is an amphoteric oxide that is capable of achieving various chemical reactions in acid and alkaline solutions. It is a high direct band gap (3.37 eV) material with a large exciton binding energy (60 meV). Its easy development and nanostructuring process make the material attractive for various applications. The performance of sensing surfaces highly relies on nanostructures to enhance their sensitivity and specificity [47,48]. A ZnO film of around 200 nm thickness embedded with an interdigitated electrode can act as a pH sensing electrode, which can sense pH variations in the range of 2–10 as reported in Ref. [47]. The study showed that the ZnO electrode exhibited an extremely sensitive response of 444 μAmM^−1^cm^−2^ accompanied by a strong linear regression value (R^2^ = 0.9304). The developed device demonstrated a measured sensitivity of 3.72 μA/pH. Recently, researchers proposed an innovative approach using an electrolyte insulator semiconductor (EIS) device based on ZnO nanorod-sensing membrane layers doped with magnesium. Incorporating magnesium (ranging from 0% to 5%) into the ZnO nanorods, the researchers were able to enhance the sensing capabilities of the pH sensor. The results demonstrated that ZnO nanorods doped with 3% magnesium exhibited remarkable sensing performance for hydrogen ions. These nanorods displayed high sensitivity (around 83.77 mV/pH), indicating the ability to accurately detect changes in pH levels. The linearity of the sensor response was excellent, with a value of 96.06%. Hysteresis, which refers to the variation in the sensor output when the pH is increased or decreased, was found to be minimal at 3 mV. Additionally, the sensor exhibited negligible drift value (0.218 mV/h) over time, indicating its long-term stability. The improved sensing characteristics of the ZnO nanorods doped with magnesium were attributed to the enhanced crystalline quality and the role of surface hydroxyl groups on the ZnO nanorods. These factors contributed to the increased sensitivity and reliable response of the sensor to pH variations. Yang, P-H. et al. demonstrated improved sensitivity by slightly modifying the ZnO composition. Doping aluminium to ZnO, they prepared AZO films on a flexible substrate by using RF magnetron sputtering and found a 1.5-fold higher sensitivity (average) to the pH range 2–10. Moreover, the detection characteristics varied depending on the doping concentration, making the sensor suitable for various biomedical detection applications involving different analytes [49,50].

**Figure 5:**
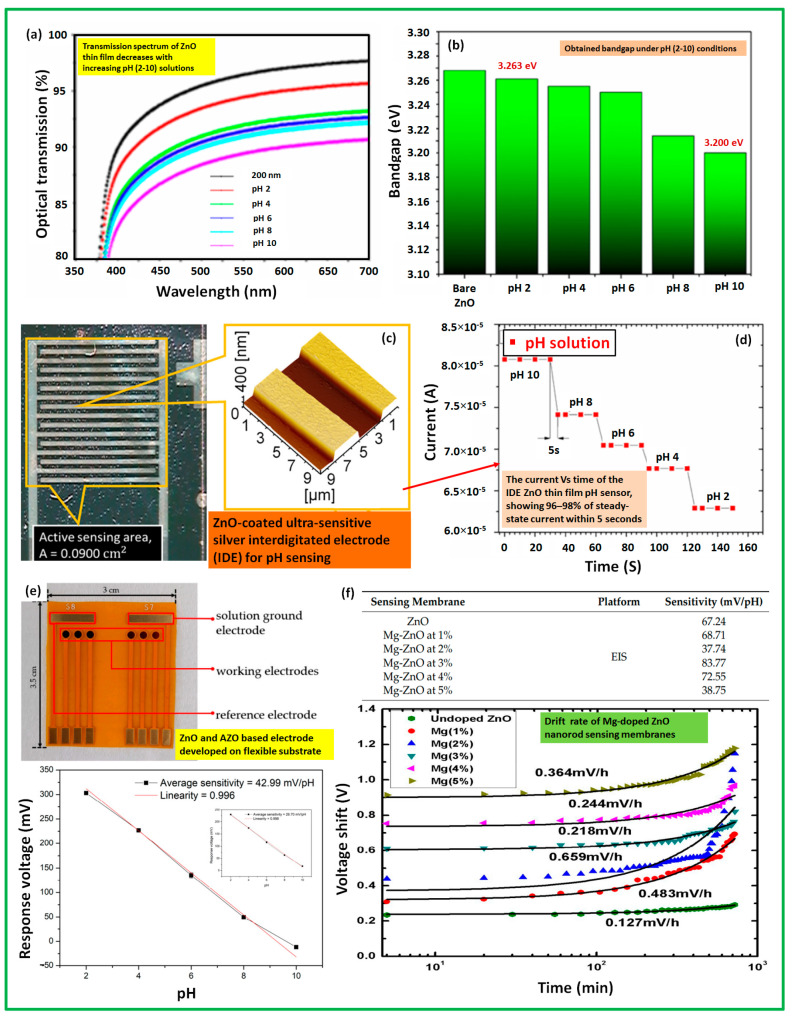
A glimpse of ZnO-based pH sensing electrode development: effects of pH on the optical properties of ZnO film (**a**), thus leading to determine the pH dependence of the bandgap of ZnO film (**b**); photolithography-assisted developed ZnO-coated ultra-sensitive silver integrated electrode (IDE), and the steady-state current response performance with different pH solutions (**c**,**d**); development of and performance comparison in terms of average sensitivity for ZnO and aluminum doped ZnO (AZO)-based pH electrodes (**e**), and the sensitivity improvement on Magnesium (Mg)-doped ZnO pH sensing electrodes (**f**) [47,50,51].

Also, the potential of ZnO suggests the suitability of using ZnO as a low-cost, high-performance, and reusable material for resistive humidity sensors. In addition, the researchers fabricated a trifunctional flexible sensor on a polyethylene terephthalate (PET) fiber surface using ZnO and ZnO/V_2_O_5_ composite layers [51]. This electrode exhibited outstanding performance as a combined temperature, glucose, and pH sensor, making it highly promising for the development of multifunctional sensors in future wearable devices. Therefore, due to its abundance, controllable surface morphology, wide resistivity range, chemical stability, and thermal stability, ZnO holds great potential for the development of high-performance and reusable pH sensors, as well as multifunctional sensors in next-generation wearable technology. Figure 5 presents a glimpse of ZnO-based pH sensor development and performance.

**Figure 6:**
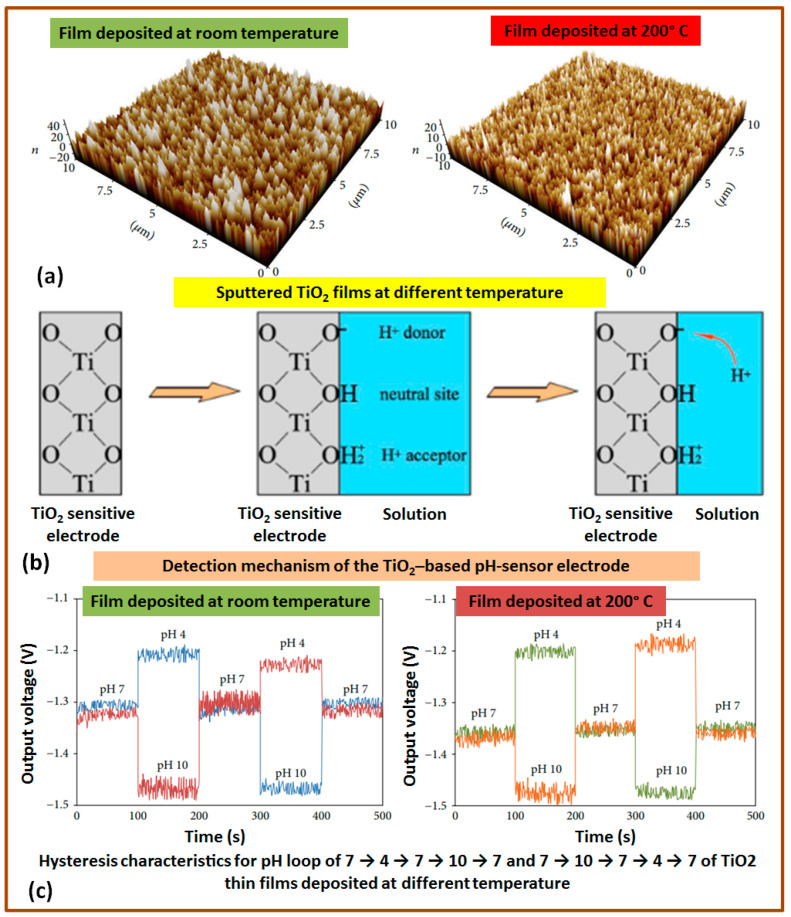
TiO_2_-based pH sensing electrodes development: microstructure evaluation of TiO_2_ films deposited at different temperatures (**a**), working principle of TiO_2_-base pH electrode (**b**), and the observed effects of deposition process parameters (such as film growth temperature) on the hysteresis characteristics of pH loop (**c**) [54,56].

There are several studies reported during the last decade about the goodness and the possibility of using physical-vapor-deposited titanium dioxide (TiO_2_) thin films for pH sensing applications. A study was found to investigate the effect of process parameters (deposition temperature and drying conditions) and composite materials on the sensing performance of TiO_2_ thin films in extended gate field-effect transistor (EGFET) pH sensors. The impact of deposition temperature on thin film properties and pH detection application was analyzed, revealing that TiO_2_ thin films sputtered at room temperature exhibited higher sensitivity compared to those deposited at high temperatures around 200 °C, while the influence of drying temperatures on the pH sensing capability of TiO_2_ thin films in bilayer structures with zinc oxide (ZnO) and semiconducting polyaniline (PANI) was found to affect the sensitivity, hysteresis, and drift characteristics of the pH sensors. However, research suggests that the optimal performance can be achieved if the ZnO/TiO_2_ thin film is dried at around 150 °C. In addition, super-hydrophilic pH-sensitive electrodes using porous TiO_2_ thin films obtained through chemical etching have also been investigated. The parameters of the etching process significantly influenced the morphology, composition, and wettability of the fabricated electrodes, affecting the pH sensing performance significantly. However, a highly selective and sensitive EGFET-pH sensor based on a composite of TiO_2_ with semiconducting PANI exhibited a super-Nernstian behavior with high sensitivity, good repeatability, low hysteresis, and low drift rates, suitable for an ideal pH sensing electrode. Overall, various factors influence the sensing performance, including deposition temperature, drying conditions, composite materials, and surface morphology. The deposition temperature and proper drying conditions (after thin film deposition) stimulates film quality by affecting crystallinity and grain size, as well as abolishing residual solvents or moisture to a certain extent and thus promoting electrical conductivity and consistent pH sensitivity, while the incorporation of composite materials results in a particular surface chemistry and catalytic capabilities, which can substantially improve the sensor’s sensitivity, reliability, and response time. Furthermore, the grain size, roughness, and porosity of the thin films can affect the amount of active surface area that impacts the sensor’s stability over time, as well as overall sensitivity (as shown in Figure 6). These fundamentals must be taken into account while designing and manufacturing metal oxide thin film pH sensors. However, the findings contribute to the understanding and design of efficient and reliable pH sensors utilizing TiO_2_ thin films as the sensing membrane [52,53,54,55,56].

In addition to all these metal oxides, physical-vapor-deposited nitrides, such as titanium nitride (TiN), silicon nitride (Si_3_N_4_), and aluminum nitride (AlN), have also been studied in the past decade to improve and develop very robust and cost-effective pH sensing electrodes/devices that offer higher thermal stability, mechanical strength, and compatibility with harsh environments [57,58,59,60]. Also, nanostructured films, comprising nanostructured metals or metal oxides, exhibit a large surface-to-volume ratio, enhancing sensitivity and electrochemical performance. When comparing these materials, metal oxides generally exhibit high pH sensitivity owing to their surface properties and charge transfer capabilities. Nitrides are suitable for pH sensing in aggressive conditions, while nanostructured films show enhanced sensitivity due to their increased surface area. Stability-wise, metal oxides demonstrate chemical resistance, nitrides exhibit corrosion resistance, and nanostructured films’ stability depends on the specific material and structure. Ultimately, material selection for pH sensing should consider the target pH range, fabrication techniques, and operating conditions to ensure optimal performance and reliability.

### 2.3. Enhancing pH Sensing Performance

It is possible to obtain enhanced pH sensing performance with thin film metal oxides by exploring new materials and employing innovative and nano-engineering approaches such as surface functionalization or texturization through substrate treatments, as the material surface and the substrate surface play a vital role in the growth of the material layers. The very latest research on the development of modified material layers, including bilayer/multilayer or composite materials, indicates the future dimension of pH sensing-related research. pH sensing could benefit from the development of new composite materials (mixing of metallic elements with either semi-metallic or non-metallic elements) as well as the development of special nano-features (nano-particles to nano-gratings) that could change the properties of the materials and thus lead to improved pH response characteristics. Material surface modification offers the increment of active surface area, specific functionalities for selectively interacting with the target pH-sensitive analyte, and protection from unwanted chemical reactions, and thus enables faster response times by endorsing efficient mass transport and tumbling diffusion boundaries. Therefore, surface engineering or texturization strategies, including self-assembled monolayers, polymeric coatings, and nanostructured surface modifications, offer promising approaches to achieve enhanced pH sensitivity. However, optimization of the surface characteristics of the metal oxide films by controlling the material layer/layers’ growth is required to achieve the tailorability of the pH sensors for specific applications, leading to improved overall performance and reliability. Very diverse research works have been conducted globally and it is not easy to summarize and showcase the beauty of all the works related to pH sensor/sensing device development. Moreover, conventional glass-electrode-based pH sensors are popular for the pH measurement. However, their mechanical fragility and lack of flexibility and bendability hamper their implementations in emerging areas such as wearable systems. Further, their performance instability at high temperatures and pressures limits their use for various industrial applications. These drawbacks of glass-electrode-based pH sensors have persuaded researchers to explore alternative methods of pH sensing. Among these, MOx-based pH sensors have attracted significant attention due to their high sensitivity (close to Nernstian response), fast response time (<10 s), long lifetime (>1 year), ease of miniaturization, stability in different atmospheres, and biocompatibility of the majority of MOx [36]. In this article, however, we have tried our best to focus on and discover the very latest updates about metal oxide thin-film-based pH sensors. The most recent research and development trends indicate that multi-structure/multilayer and composite-type material matrices are gaining more attention in terms of the future development of pH sensing devices; some examples are given in Table 1. 

## 3. Applications and Future Perspectives

pH sensing plays a crucial role in various fields, including environmental monitoring, biotechnology, and medical diagnostics. pH sensing helps to assess acidity or alkalinity levels, which can have a significant impact on aquatic life and ecological systems. pH sensors are also used in soil analysis to determine optimal pH levels for agriculture and environmental management. In terms of biotechnological applications such as fermentation processes, enzyme activity assays, and cell culture systems, maintaining proper pH levels is critical for optimizing biochemical reactions and cell growth. pH sensors enable real-time monitoring and control, ensuring the desired pH conditions are also suitable for various medical diagnostics. For example, pH capsules are in use for non-invasive monitoring of pH levels in the gastrointestinal tract, aiding in the diagnosis of acid reflux and other digestive disorders. pH sensors are also utilized in biosensing platforms for detecting pH changes associated with diseases or physiological abnormalities. Table 2 summarizes the currently available pH sensing devices, their application areas, and their limitations.

PVD thin films are footing a momentous perspective for impending pH sensing technologies due to their exceptional possessions and fabrication flexibility when controlling film growths (thickness, material stoichiometry, and morphology). The development of advanced pH sensors/sensing devices is possible only with thin films with tailored properties and enhanced sensing capabilities, thus opening the space for thin films to be integrated into microfluidic devices, lab-on-a-chip systems, and wearable sensors, enabling miniaturization, portability, and multiplexing of pH sensing devices. However, the development of highly selective and stable pH-sensitive materials is still considered a great challenge as the thin films should be specific to hydrogen ions while being insensitive to other ions or environmental factors. The steady and long-term performance of pH sensors is desirable for accurate and reliable measurements over extended periods. In addition, the miniaturization of pH sensors for point-of-care and in vivo applications is an active area of research. Integration of pH sensors with wireless communication systems, and biocompatible materials can enable real-time monitoring and non-invasive pH measurements in biomedical settings. The improvement of sensing mechanisms via exploring modern technological advancement for material synthesis and development such as composite materials and multilayer structures development, surface plasmon resonance, surface texturization, and nanostructure functions can further provide new opportunities for pH sensing technologies. Furthermore, due to their distinctive features and performance traits, metal oxide thin film pH sensors have a large amount of promise in energy production and energy saving applications as well. These sensors can help to streamline procedures, increase productivity, and lower energy usage in a variety of contexts. Metal oxide thin film pH sensors provide precise monitoring of an electrolyte’s pH level in energy-generating applications like fuel cells and batteries. These sensors support electrochemical process optimization by maintaining the ideal pH range, which enhances energy conversion efficiency and lengthens device longevity. Also, metal oxide thin film pH sensors are effective for various industrial monitoring and water treatment and management applications. They make it possible to precisely monitor the pH in cooling towers, boilers, and wastewater treatment systems, enabling effective chemical dosing management and reducing energy losses brought on by corrosion, scaling, or inefficient chemical reactions. Additionally, metal oxide thin film pH sensors can also be used to monitor and regulate the pH level at different phases of the production process in renewable energy systems like the fabrication of solar cells and modules. This guarantees the effectiveness and integrity of the production process, resulting in improved energy conversion and less waste. Furthermore, the integration of advanced data analysis techniques, including machine learning and data fusion, can help extract valuable information from pH sensor outputs and enable more sophisticated pH monitoring and control systems.

## 4. Conclusions

pH sensing is of the utmost importance in various fields such as biotechnology, biomedicine, environmental monitoring, and industrial processes. Physical vapor deposition (PVD) thin films have emerged as promising candidates for pH sensing applications due to their exceptional properties and fabrication flexibility. The history of PVD metal oxide thin films reflects that in the last decade, some metal oxides, including ruthenium oxide (RuO_2_), zinc oxide (ZnO), and titanium dioxide (TiO_2_), and some metal nitrides, have been extensively studied for their pH sensing capabilities. PVD thin films offer advantages such as high sensitivity, compatibility with different substrates, precise control over material properties, and long-term stability. Surface modification techniques and nano-structuring approaches further enhance pH sensitivity. However, challenges related to deposition thickness, film uniformity, and material choice need to be addressed. Ongoing research focuses on optimizing deposition techniques and exploring innovative approaches to overcome these challenges. Future directions include the development of composite materials, surface engineering techniques, and the integration of thin films into miniaturized devices such as wearable sensors and lab-on-a-chip systems. These advancements hold promise for real-time pH monitoring in biomedical applications and energy-saving processes. Overall, PVD thin films offer great potential for advancing pH sensing technologies and addressing various application needs. However, the focus of this article underlies as follows:

- The article highlights the significant advancements in physical-vapor-deposited (PVD) metal oxide thin films for pH sensing applications over the last decade. PVD techniques such as sputtering, evaporation, and ion beam deposition have been explored to fabricate thin films with tailored properties for high-performance pH sensors.
- The incorporation of magnesium into ZnO nanorods has been found to enhance the sensing capabilities of pH sensors, with ZnO nanorods doped with magnesium exhibiting remarkable sensing performance for hydrogen ions.
- The use of multi-structures, multilayer, and composite-type material matrices is gaining attention for the future development of pH sensing devices, as they offer improved performance and fine-tuning functionality.

## Figures and Tables

**Table 1:** Summary of accomplished research work related to PVD thin film materials (single layer, multilayer, or composites) for pH detection applications in the past decade.

Research Group/Publication Place	Material Type	Fabrication Technique	Major Findings	Remarks
Ameri, S.K. et al., *Analytical Chimica Acta* [61]	Graphene and HfO_2_	Combined processes including CVD and Sputtering	3D liquid-gated graphene transistor based on highly sensitive pH sensor development. pH sensitivity was found to be very high, (71 ± 7) mV/pH, exceeding the Nernst limit.	The HfO_2_ layer was found to influence the pH measurement. Further investigation and study are required for the possible variations in blood background.
Jovic, M.J.C. et al., *Journal of Electroanalytical Chemistry* [62]	Iridium oxide (IrO_2_)	-	Excellent pH sensitivity, response time, and reproducibility of the pH electrodes were observed. The recorded pH responses were around 59 mV/pH.	An innovative method to develop a scalable, patterned, and flexible pH sensor with enhanced responses.
Krishna, B.G. et al., IEEE International WIE Conference on Electrical and Computer Engineering (WIECON-ECE) [63]	FeTiO_3_/SiO_2_ Composite	Bionanotechnology	Glucose molecule detection in the blood is performed by evaluating the V-pH characteristics of the biosensor.	
Rasheed, H.S. et al., *J. Electronic Materials* [64]	ZnO and Pd	RF/DC sputtering	Multilayer ZnO/Pd/ZnO thin film structure can reduce the sheet resistance and increase the conductivity of ZnO sensing membranes. The measured pH sensitivity was 40 μA/pH and 52 mV/pH within the pH range from 2 to 12.	Applicable for various applications, such as pH sensors and biosensors; further examination is required.
Sun, K.G. et al., *ACS Applied Materials and Interface* [65]	Al_2_O_3_ and ZnO	Atomic layer deposition (ALD)	Highly selective deposition of Al_2_O_3_ over ZnO can protect the ZnO layer in alkaline aqueous solutions with pHs between about 9 and 12.	
Sharma, N. et al., *Mater. Res. Express* [66]	Ta_2_O_5_	E-beam evaporation	Very reliable and reusable pH sensing electrodes were developed. The reusable response time was less than 5 s.	Ta_2_O_5_ thin films were found very promising for pH sensing electrode material.
Xu, K. et al., *IEEE Sensors Journal* [67]	Sb/Sb_2_O_3_ metal/metal oxide	Magnetron sputtering	pH sensor heat-treated at 200 °C exhibited the best sensitivity (74.0 mV/pH) performance. Less than 3 s response time was observed.	Suitable for pH detection in soilless culture.
Yang. J. et al., *ACS Sensors* [68]	IrO_2_, and Graphene oxide (GO)		IrO_2_-reduced GO exhibited super-Nernstian linear responses from pH 2 to 12. A miniaturized and portable pH device has been fabricated and performance-tested.	Possible to prepare low-cost and convenient pH strips.
Yoo, T. et al., *J. Korean Physical Society* [69]	InGaZnO	ALD	IGZO transistor-based pH sensors in the aqueous medium have been reported.	
Zulkefle, M.A. et al., *UTM Journal Teknologi* [70]	ZnO and TiO_2_	Sol-gel spin-coating method	The thin films were tested as a pH sensing membrane for the extended-gate field effect transistor (EGFET) sensor. TiO_2_ thin film has higher sensitivity with better linearity compared to pure ZnO film.	TiO_2_ is preferable for sensing membrane in an EGFET pH sensor.
Hajjaji, A. et al., *Sensor Letters* [71]	Cr and TiO_2_	RF magnetron sputtering	Cr-doped dependent band-gap variation was observed.	Mostly suitable for gas sensing.

**Table 2:** Types of pH sensing devices, applications, and their pros and cons.

pH Sensing Device	Description	Application	Cost	Sample Size	Portability
pH Electrodes	Glass electrode and reference electrode required to measure pH	Industrial, laboratory, research	Moderate	Small to large	Portable/Non-portable
pH Test Strips	Paper strips with color-changing indicators	Quick qualitative assessments	Low	Small	Highly portable
pH Meters	Portable or benchtop devices with a digital display	Laboratories, research facilities, industrial use	High	Small to large	Portable (depends on the device)
pH Probes	Robust electrodes designed for specific applications	Solid samples, penetration measurements	Moderate to high	Varies	Portable (depends on the device)
pH Capsules	Small, disposable, wireless sensors for non-invasive pH measurements in the human body	Medical applications (gastrointestinal monitoring)	High	Small	Highly portable
pH Imaging Systems	Utilize fluorescent dyes/indicators for visualizing and mapping pH variations in biological samples	Biological research, imaging	High	Varies	Non-portable (benchtop equipment)
Microfluidic pH Sensors	Miniaturized devices with microfluidic channels	Biomedical and environmental monitoring	Moderate to high	Small	Portable (depends on the device)

## Data Availability

Not applicable.
